# Galangin Inhibits Thrombin-Induced MMP-9 Expression in SK-N-SH Cells via Protein Kinase-Dependent NF-κB Phosphorylation

**DOI:** 10.3390/ijms19124084

**Published:** 2018-12-17

**Authors:** Chien-Chung Yang, Chih-Chung Lin, Li-Der Hsiao, Chuen-Mao Yang

**Affiliations:** 1 Department of Traditional Chinese Medicine, Chang Gung Memorial Hospital at Tao-Yuan, Kwei-San, Tao-Yuan 333, Taiwan; r55161@cgmh.org.tw; 2 School of Traditional Chinese Medicine, College of Medicine, Chang Gung University, Kwei-San, Tao-Yuan 333, Taiwan; 3 Department of Anesthetics, Chang Gung Memorial Hospital at Linkuo, and College of Medicine, Chang Gung University, Kwei-San, Tao-Yuan 333, Taiwan; chihchung@adm.cgmh.org.tw (C.-C.L.); lidesiao@livemail.tw (L.-D.H.); 4 Department of Physiology and Pharmacology and Health Ageing Research Center, College of Medicine, Chang Gung University, Kwei-San, Tao-Yuan 333, Taiwan; 5 Research Center for Industry of Human Ecology and Graduate Institute of Health Industry Technology, Chang Gung University of Science and Technology, Tao-Yuan 333, Taiwan

**Keywords:** galangin, thrombin, MMP-9, protein kinases, signaling pathways, transcription factors

## Abstract

Galangin, a member of the flavonol compounds of the flavonoids, could exert anti-inflammatory effects in various cell types. It has been used for the treatment of arthritis, airway inflammation, stroke, and cognitive impairment. Thrombin, one of the regulators of matrix metalloproteinase (MMPs), has been known as a vital factor of physiological and pathological processes, including cell migration, the blood–brain barrier breakdown, brain edema formation, neuroinflammation, and neuronal death. MMP-9 especially may contribute to neurodegenerative diseases. However, the effect of galangin in combating thrombin-induced MMP-9 expression is not well understood in neurons. Therefore, we attempted to explore the molecular mechanisms by which galangin inhibited MMP-9 expression and cell migration induced by thrombin in SK-N-SH cells (a human neuroblastoma cell line). Gelatin zymography, western blot, real-time PCR, and cell migration assay were used to elucidate the inhibitory effects of galangin on the thrmbin-mediated responses. The results showed that galangin markedly attenuated the thrombin-stimulated phosphorylation of proto-oncogene tyrosine-protein kinase (c-Src), proline-rich tyrosine kinase 2 (Pyk2), protein kinase C (PKC)α/β/δ, protein kinase B (Akt), mammalian target of rapamycin (mTOR), p42/p44 mitogen-activated protein kinase (MAPK), Jun amino-terminal kinases (JNK)1/2, p38 MAPK, forkhead box protein O1 (FoxO1), p65, and c-Jun and suppressed MMP-9 expression and cell migration in SK-N-SH cells. Our results concluded that galangin blocked the thrombin-induced MMP-9 expression in SK-N-SH cells via inhibiting c-Src, Pyk2, PKCα/βII/δ, Akt, mTOR, p42/p44 MAPK, JNK1/2, p38 MAPK, FoxO1, c-Jun, and p65 phosphorylation and ultimately attenuated cell migration. Therefore, galangin may be a potential candidate for the management of brain inflammatory diseases.

## 1. Introduction

Matrix metalloproteinases (MMPs), a series of zinc-dependent endopeptidases, consist of more than 20 members, including the MMPs of humans and other species. They are typically secreted or anchored to the cell surface; thereby, they can catalyze the degradation of proteins within the secretory pathway or the extracellular matrix (ECM). Therefore, MMPs may initiate the breakdown of the blood–brain barrier (BBB) allowing the entry of immune cells and autoreactive lymphocytes that may locally secrete TSP-1 (granzyme A), tryptase, and other serine proteases in the brain tissues with inflammation [1]. Several reports have suggested that demyelination, inflammation, and neurotoxicity in many central nervous system (CNS) diseases, such as multiple sclerosis, stroke, CNS infection, and CNS degeneration, are mediated through the upregulation of MMPs [2,3,4]. Actually, several lines of evidence have shown that the inflammatory responses induced by diverse brain insults lead to the abnormal expression and activation of MMP-9, which can degrade the ECM, resulting in the disruption of the BBB, preventing normal cell signaling and eventually leading to cell death [5,6]. It has been shown for decades that the upregulation of MMP-9 has played a vital role in neuroinflammatory and neurodegenerative disorders [7,8]. These links have been made by the finding of BBB damage leading to cerebral edema, excitotoxicity, neuronal damage, apoptosis, and hemorrhagic transformation by MMP-9 in brain insults [9,10,11]. Furthermore, in a mouse model of middle cerebral artery occlusion, using a fibrin-rich clot demonstrated that the inhibition of MMPs by SB-3CT, a selective inhibitor of gelatinases, showed beneficial effects by attenuating infarct volume and ameliorating neurobehavioral outcomes [12,13]. As a consequence, it can be speculated that adjuvant intervention with gelatinases inhibitors might diminish the disease severity and brain injury by stabilizing the BBB in cases of acute stages of stroke or demyelination disorder.

Thrombin plays vital roles in the regulation of numerous physiological and pathological processes, such as vascular tone, neuroinflammation, atherogenesis, thrombosis, and neurodegeneration. High levels of thrombin may have detrimental effects, as they can cause neuron and astrocyte apoptosis, disrupt the BBB after intracerebral hemorrhage, and induce brain edema [14]. Moreover, high levels of thrombin also participate in the pathological process in Alzheimer’s disease (AD) patients [15] and in stroke models [16]. Moreover, thrombin has been revealed to induce MMP-9 production related to pathological processes in various cell types [17,18,19]. In our previous studies, thrombin was shown to induce MMP-9 expression via various signaling components in SK-N-SH cells [20,21]. In the clinic, traditional Chinese medicines rich in flavonoids have been used to treat inflammatory diseases and prevent illnesses. Galangin (3,5,7-trihydroxy-2-phenyl-4H-1-benzopyran-4-one) is a member of the flavonol compounds of the flavonoids. It exists in high concentrations in *Alpinia officinarum* and has been used as a herbal medicine for a variety of diseases. Therefore, the aim of the present study was to evaluate whether galangin inhibited thrombin-induced MMP-9 expression and cell migration in human SK-N-SH cells.

It has been demonstrated that galangin has anti-inflammatory, anti-oxidant, antimutagenic, anticlastogenic, metabolic enzyme modulating, bactericidal, and anti-fibrotic activities [22] in various disorders, such as collagen-induced arthritis and ovalbumin-induced airway inflammation via inhibiting nuclear factor-κB (NF-κB) signaling [23,24]. Recent evidence indicates that galangin has therapeutic potential in some neuroinflammatory and neurodegenerative disorders, such as stroke and cognitive impairment [25,26,27,28]. Moreover, galangin suppresses phorbol-12-myristate-13-acetate-induced MMP-9 expression by blocking the activation of the NF-κB- and activator protein 1 (AP-1)-dependent pathways in human fibrosarcoma HT-1080 cells [29]. These results suggest that galangin acts as one of the inhibitors that attenuate thrombin-mediated responses [30]; thereby, it can be a potential intervention for the management of brain diseases. Further, experiments were performed to dissect the detailed molecular mechanisms by which galangin attenuates thrombin-induced MMP-9 expression in human SK-N-SH cells. Therefore, we further evaluated whether galangin (GLG) attenuates the thrombin-stimulated activation of protein kinases, including non-receptor tyrosine receptor kinases (nRTKs), PKCs, Akt, mTOR, MAPKs, and transcription factors, such as NF-κB, AP-1, and forkhead box protein O1 (FoxO1), in human SK-N-SH cells.

## 2. Results

### 2.1. Galangin Attenuates Thrombin-Induced MMP-9 Expression and Cell Migration

**Figure 1:**
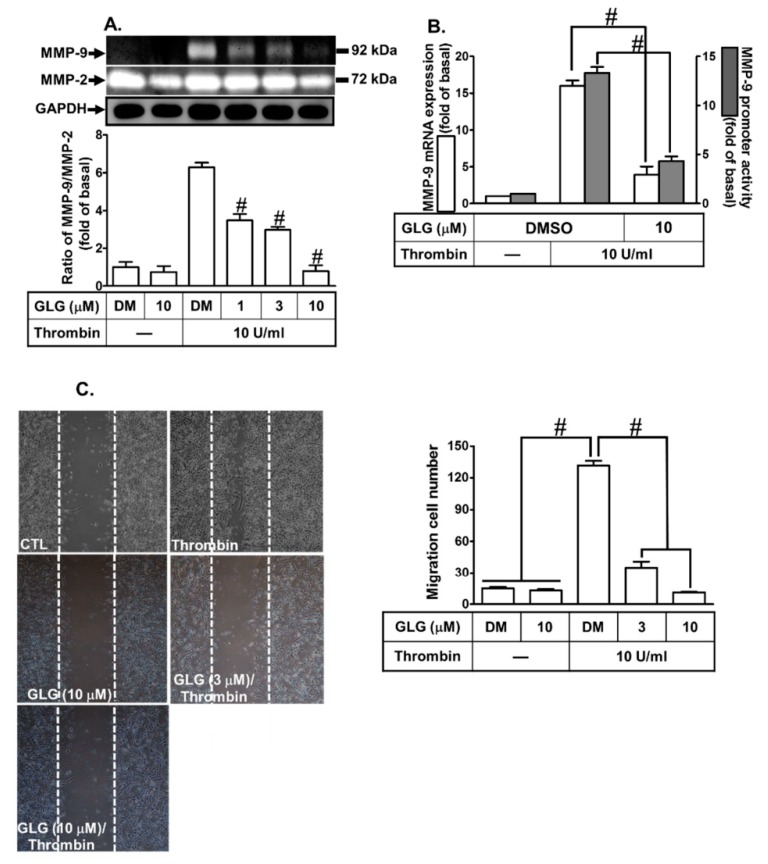
Galangin (GLG) reduces thrombin-induced pro-form (pro) MMP-9 expression and cell migration in SK-N-SH cells. (**A**) The cells were pretreated with galangin (1, 3, 10 μM) for 1 h and then incubated with 10 U/mL thrombin for 16 h. The conditioned cell culture media were collected to measure the MMP-9 expression by gelatin zymography. The activity of proMMP-9 was normalized to that of MMP-2. The cell lysates were analyzed by western blot to determine the expression of glyceraldehyde-3-phosphate dehydrogenase (GAPDH), serving as the marker for the cell viability during these treatments. (**B**) The cells were pretreated with galangin (10 μM) for 1 h and then incubated with 10 U/mL thrombin for 16 h (mRNA expression) or 24 h (promoter activity). The mRNA expression and promoter activity of MMP-9 were determined by real-time PCR and promoter assay, respectively. (**C**) The cells were plated on 6-well culture plates, grown to confluence, and then starved with serum-free medium for 24 h. At that point, the cells were pretreated with galangin (3 or 10 μM) for 1 h and then incubated with thrombin for 48 h. Images of the migratory cells were taken at the indicated conditions. The data are expressed as mean ± SEM of three independent experiments. ^#^ *p* < 0.01, as compared with the cells exposed to thrombin alone. −: no treatment with Thrombin; DM: DMSO (dimethyl sulfoxide).

We evaluated the effects of galangin on thrombin-induced MMP-9 expression. SK-N-SH cells were pretreated with galangin for 1 h and then incubated with thrombin (10 U/mL) for the indicated time intervals (16 h for protein and RNA, 24 h for promoter activity, and 48 h for cell migration). As shown in Figure 1A, pretreatment with galangin at the indicated dosage significantly reduced the thrombin-induced MMP-9 protein level, determined by gelatin zymography. In addition, pretreatment with galangin (10 μM) for 1 h also attenuated the thrombin-induced MMP-9 mRNA level and promoter activity, respectively (Figure 1B). Furthermore, to explore the inhibitory effect of galangin on the functional activity of MMP-9, we evaluated the effect of galangin on the cell migration of SK-N-SH cells challenged with thrombin. The SK-N-SH cell migration was observed 48 h after the treatment with thrombin in the absence or presence of galangin (3 or 10 μM). These data showed that the galangin reduced the migratory cell number of the thrombin-induced SK-N-SH cell migration in a concentration-dependent manner (Figure 1C). These results suggested that galangin inhibits the thrombin-induced MMP-9 expression associated with cell migration in SK-N-SH cells.

### 2.2. Galangin Attenuates Thrombin-Induced Non-Receptor Tyrosine Kinase (nRTK) Activity

**Figure 2:**
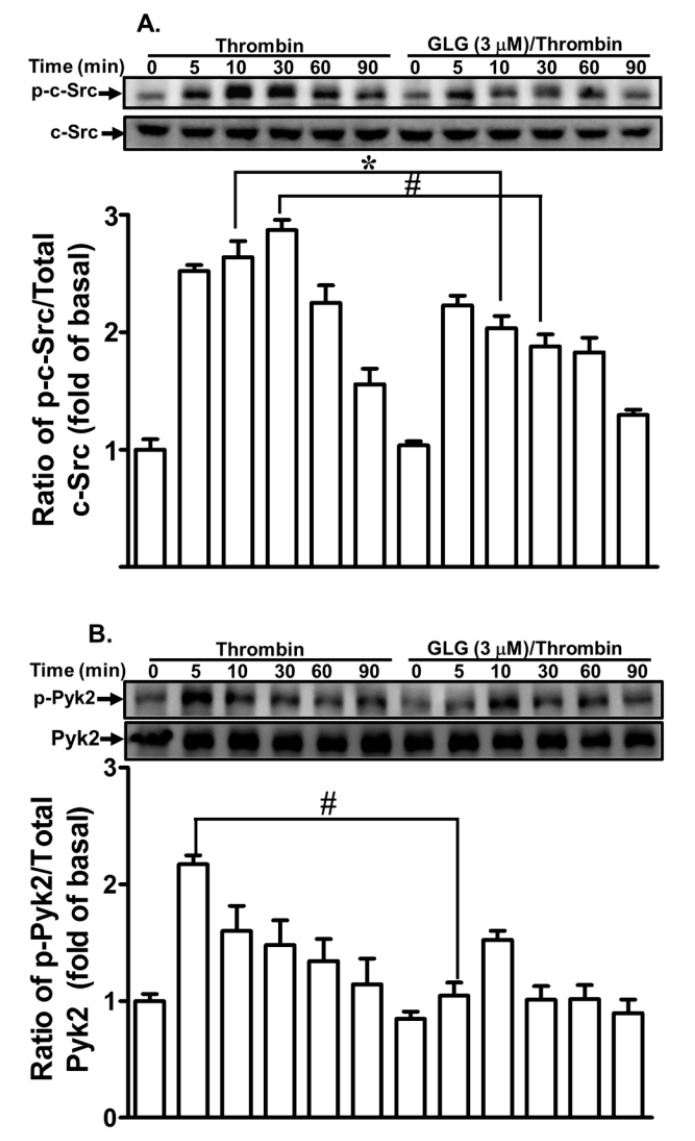
Galangin inhibits the thrombin-induced phosphorylation of proto-oncogene tyrosine-protein kinase (c-Src), proline-rich tyrosine kinase 2 (Pyk2). The cells were pretreated with galangin (3 μM) for 1 h and then incubated with thrombin (10 U/mL) for the indicated time intervals. The levels of (**A**) c-Src and (**B**) Pyk2 phosphorylation and their respective protein levels were determined by western blot. The data are expressed as mean ± SEM of three independent experiments. * *p* < 0.05; ^#^ *p* < 0.01, as compared with the cells exposed to thrombin alone.

Several studies have uncovered that several nRTKs, including c-Src and Pyk2, can regulate MMP-9 expression in various cell types [18,31,32]. Our previous results also found that c-Src and Pyk2 are involved in the thrombin-induced MMP-9 expression in SK-N-SH cells [20]. Therefore, we further explored whether galangin can attenuate the thrombin-stimulated activation of nRTKs in SK-N-SH cells. We discovered that pretreatment with galangin (3 μM) reduced the phosphorylation of c-Src (Figure 2A) and Pyk2 (Figure 2B) stimulated by thrombin. These results suggested that galangin reduces thrombin-induced MMP-9 expression via attenuating the c-Src and Pyk2-dependent pathways in SK-N-SH cells.

### 2.3. Galangin Reduces Thrombin-Stimulated Phosphorylation of PKCs

**Figure 3:**
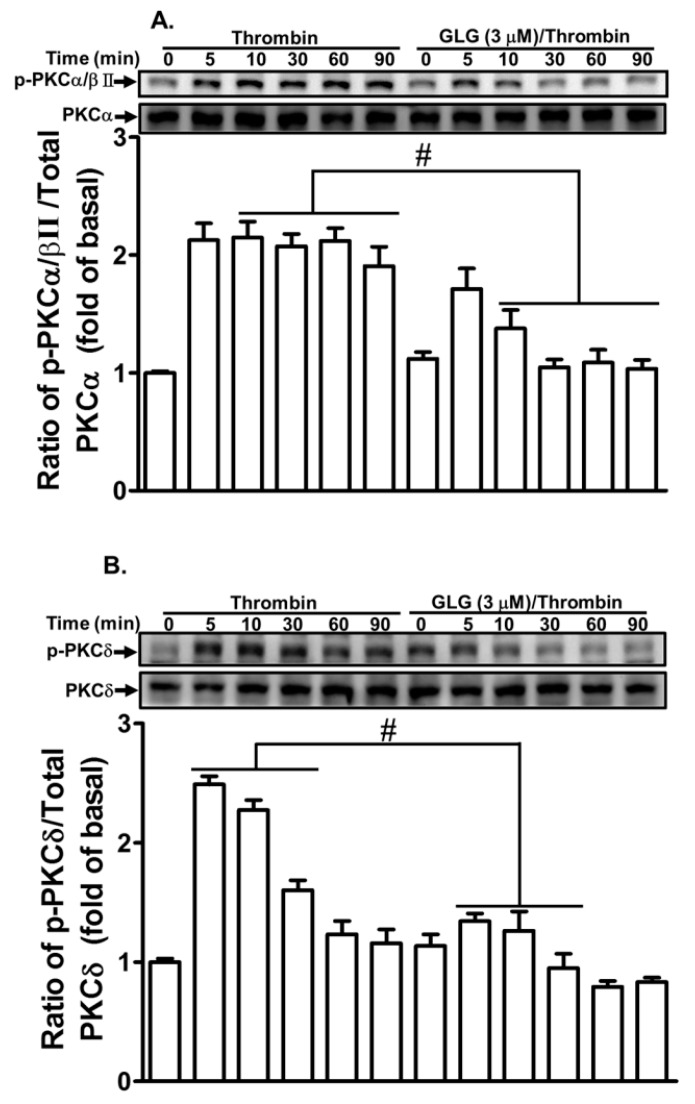
Galangin inhibits thrombin-induced phosphorylation of protein kinase C (PKC)α/βII and δ. The cells were pretreated with galangin (3 μM) for 1 h and then incubated with thrombin (10 U/mL) for the indicated time intervals. The levels of (**A**) PKCα/βII and (**B**) PKCδ phosphorylation and their respective protein levels were determined by western blot. The data are expressed as mean ± SEM of three independent experiments. * *p* < 0.05; ^#^ *p* < 0.01, as compared with the cells exposed to thrombin alone.

Recent reports have shown that PKC-dependent pathways contribute to MMP-9 expression in various cell types [18,33,34]. Our recent study also revealed that PKCs play a key role in the thrombin-induced MMP-9 expression in SK-N-SH cells [21]. Therefore, we further evaluated the effect of galangin on the thrombin-stimulated activation of PKCs. We demonstrated that pretreatment with galangin (3 μM) attenuated the thrombin-stimulated phosphorylation of PKCα/βII or δ in SK-N-SH cells (Figure 3). These results indicated that galangin reduces the thrombin-induced MMP-9 expression by suppressing PKCα/βII or δ activation in SK-N-SH cells.

### 2.4. Galangin Reduces Thrombin-Stimulated Phosphorylation of Mammalian Target of Rapamycin (mTOR)

**Figure 4:**
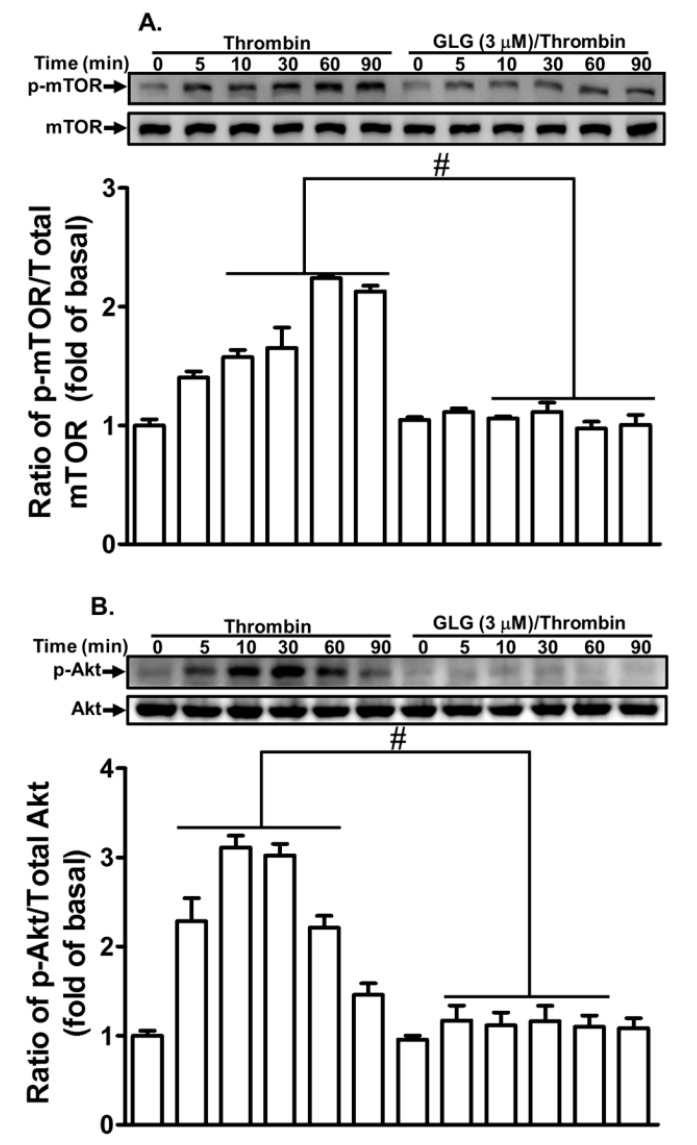
Galangin inhibits thrombin-induced phosphorylation of Akt and mammalian target of rapamycin (mTOR). The cells were pretreated with galangin (3 μM) for 1 h and then challenged with thrombin (10 U/mL) for the indicated time intervals. The levels of (**A**) mTOR and (**B**) Akt phosphorylation and their respective protein levels were determined by western blot. The data are expressed as mean ± SEM of three independent experiments. * *p* < 0.05; ^#^ *p* < 0.01, as compared with the cells exposed to thrombin alone.

Several reports have found that mTOR is involved in MMP-9 expression in various cell types [35,36,37]. Our previous study also found that PI3K/Akt are key players in the thrombin-induced MMP-9 expression in SK-N-SH cells [20]. Therefore, we further explored whether galangin can attenuate the thrombin-induced mTOR-dependent pathway in SK-N-SH cells. We determined that pretreatment with galangin (3 μM) reduced the phosphorylation of mTOR (Figure 4A) and Akt (Figure 4B) stimulated by thrombin. These results suggested that galangin reduces the thrombin-induced MMP-9 expression via attenuating the activation of mTOR and Akt-dependent pathways in SK-N-SH cells.

### 2.5. Galangin Inhibits Thrombin-Stimulated MAPKs Activity

**Figure 5:**
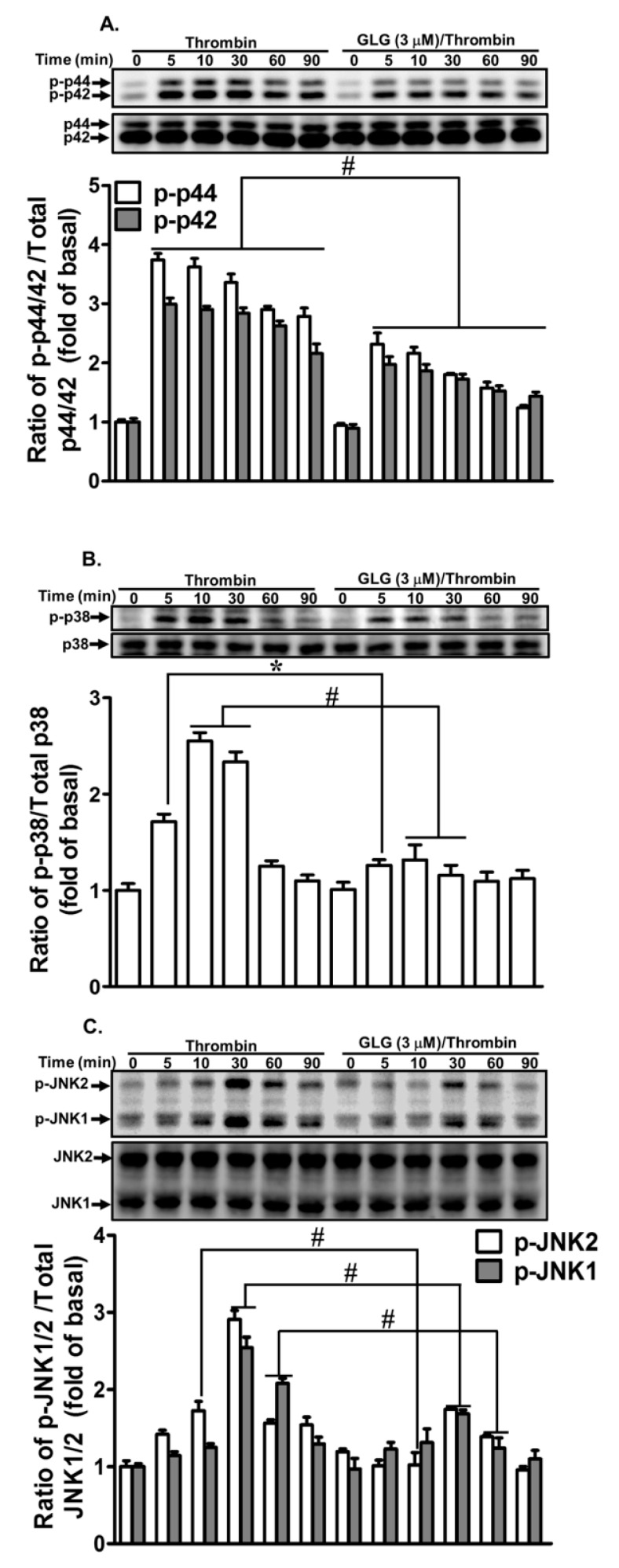
Galangin inhibits thrombin-induced phosphorylation of p42/p44 mitogen-activated protein kinase (MAPK), p38 MAPK, and Jun amino-terminal kinases (JNK)1/2. The cells were pretreated with galangin (3 μM) for 1 h and then challenged with thrombin (10 U/mL) for the indicated time intervals. The levels of (**A**) p42/p44 MAPK, (**B**) p38 MAPK, and (**C**) JNK1/2 phosphorylation and their respective protein levels were determined by western blot. The data are expressed as mean ± SEM of three independent experiments. * *p* < 0.05; ^#^ *p* < 0.01, as compared with the cells exposed to thrombin alone.

Recent reports have demonstrated that MAPK-dependent pathways are involved in thrombin-induced MMP-9 expression in various cell types [38,39,40]. Our recent data have also revealed that MAPKs participate in the thrombin-induced MMP-9 expression in SK-N-SH cells [20,21]. Therefore, we further explored whether galangin reduced the MAPKs-dependent pathways associated with the attenuation of thrombin-induced MMP-9 expression. We found that pretreatment with galangin (3 μM) attenuated the thrombin-stimulated phosphorylation of p44/p42 MAPK (Figure 5A), p38 MAPK (Figure 5B), and JNK1/2 (Figure 5C) in SK-N-SH cells. These results indicated that galangin-reduced thrombin-induced MMP-9 expression is mediated via suppressing the MAPKs activation in SK-N-SH cells.

### 2.6. Galangin Inhibits Thrombin-Stimulated Transcription Factor Activation

**Figure 6:**
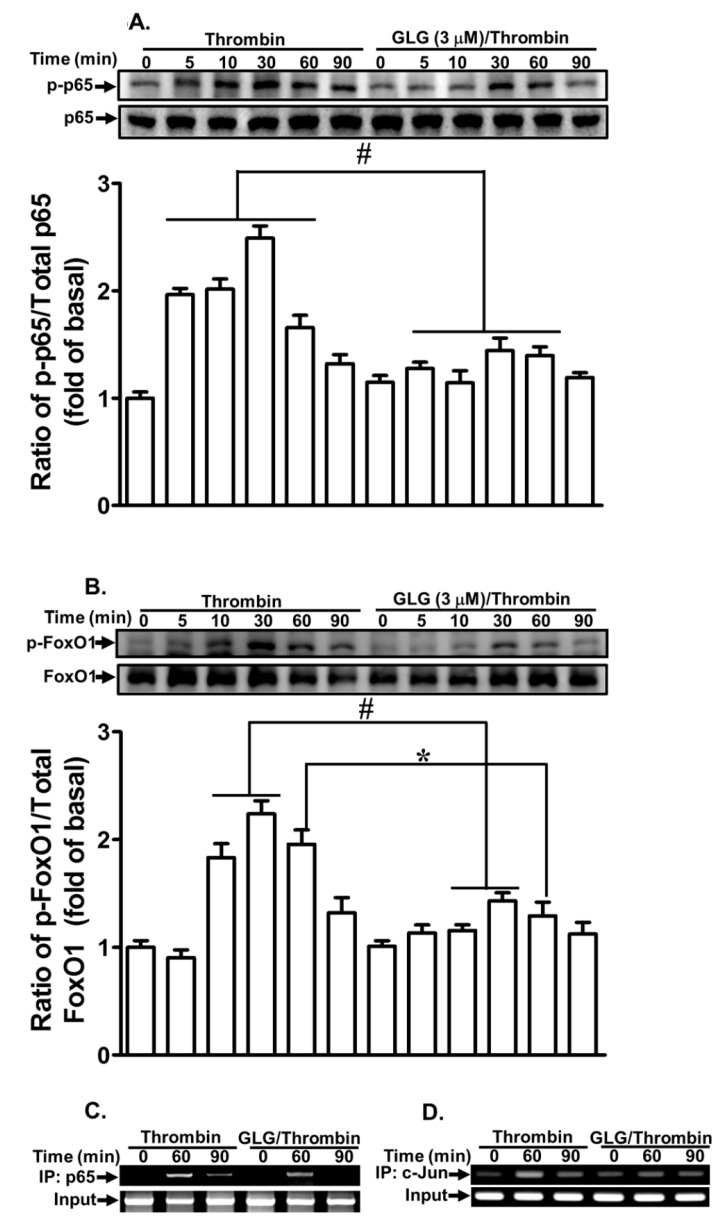
Galangin reduces thrombin-induced proMMP-9 expression via inhibiting nuclear factor-kappaB (NF-κB), activator protein 1 (AP-1), and forkhead box protein O1 (FoxO1). The cells were pretreated with galangin (3 μM) for 1 h and then challenged with thrombin (10 U/mL) for the indicated time intervals. The levels of (**A**) p65 and (**B**) FoxO1 phosphorylation and their respective protein levels were determined by western blot. (**C**, **D**) The cells were pretreated with/without galangin (3 μM) for 1 h and then incubated with thrombin (10 U/mL) for the indicated time intervals, chromatin immunoprecipitation (ChIP) assay was performed by using the antibodies-p65 (**C**) and c-Jun (**D**), respectively. The data are expressed as mean ± SEM of three independent experiments. * *p* < 0.05; ^#^ *p* < 0.01, as compared with the cells exposed to thrombin alone.

Several transcription factors, such as NF-κB, AP-1, and FoxO1, have been revealed to be involved in MMP-9 expression in various cell types [20,41,42,43]. Our previous studies also showed that NF-κB and AP-1 contribute to the thrombin-induced MMP-9 expression in SK-N-SH cells [20,21]. Here, we explored whether galangin blocked the transcription factors involved in the thrombin-induced MMP-9 expression in SK-N-SH cells. The NF-κB, AP-1, and FoxO1-dependent pathways were detected by western blot and chromatin immunoprecipitation (ChIP) assays. Our results showed that pretreatment with galangin significantly reduced the thrombin-stimulated phosphorylation of p65 (Figure 6A) and FoxO1 (Figure 6B). The interactions of p65 (Figure 6C) and c-Jun (Figure 6D) with MMP-9 promoter were blocked by galangin in SK-N-SH cells challenged by thrombin. These results indicated that galangin reduces the thrombin-induced MMP-9 expression by suppressing the activation of the transcription factors, including NF-κB, AP-1, and FoxO1 in SK-N-SH cells.

## 3. Discussion

**Figure 7:**
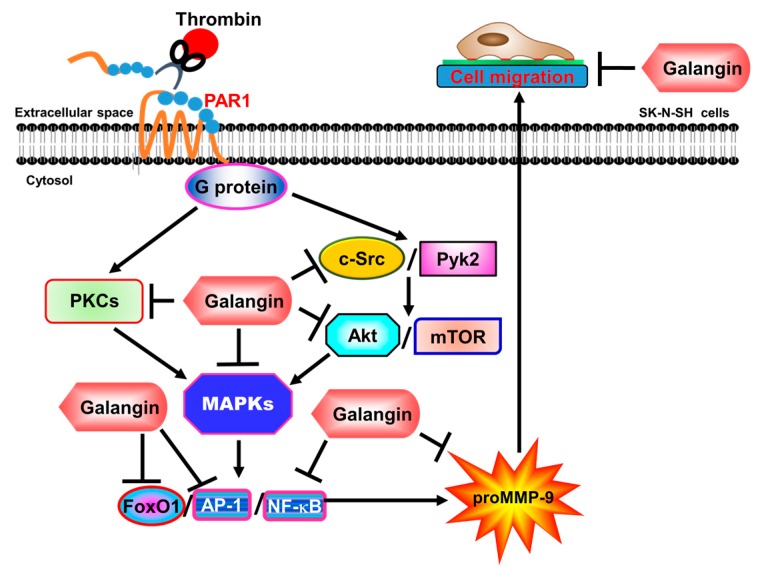
The schematic signaling pathways involved in galangin inhibiting the MMP-9 expression in SK-N-SH cells challenged with thrombin. Galangin inhibits MMP-9 expression by attenuating c-Src, Pyk2, PKCα/β/δ, Akt, mTOR, p42/p44 MAPK, p38 MAPK, and JNK1/2 phosphorylation to decrease NF-κB, AP-1, and FoxO1 activation and ultimately reduces SK-N-SH cell migration.

Among MMPs, uncontrolled MMP-9 expression and activity lead to the pathological effects of brain disorders, such as stroke, brain tumor, and AD [7,44,45]. Thrombin also participates in neuroinflammatory reactions in these brain disorders [46], suggesting its role in neurodegenerative processes. Therefore, the inhibitors of thrombin and MMP-9 have therapeutic potential in neuroinflammatory diseases [13,30]. Flavonoids are a group of polyphenolic compounds and exert many biological activities, including anti-inflammatory, antioxidant, and antitumor capabilities. These compounds have been elucidated to inhibit two members of the MMP family, MMP-2 and MMP-9, and thrombin activity [30,47]. Here, we evaluated whether galangin had inhibitory effects on the thrombin-induced MMP-9 expression and signaling components in SK-N-SH cells. Therefore, the concentrations of galangin (up to 10 µM) used in this study had no effect on the cell viability and allowed us to evaluate how galangin attenuated the thrombin-stimulated activation of the protein kinases and transcription factors in human SK-N-SH cells. However, higher dosages (greater than 10 µM) of galangin could induce apoptosis (Supplementary Figure S1). Our results elucidated that in SK-N-SH cells, galangin blocked the thrombin-induced MMP-9 expression and cell migration via inhibiting the activation of PKCs, Akt, mTOR, c-Src, Pyk2, p38 MAPK, p44/p42 MAPK, JNK1/2-dependent FoxO1, AP-1, and p65 signaling pathways (Figure 7).

To the best of our knowledge, this is the first report showing that galangin has anti-inflammatory effects on SK-N-SH cells by attenuating the thrombin-induced MMP-9 expression and cell migration. Our previous studies and others have reported that thrombin-stimulated MMP-9 expression is mediated via c-Src, Pyk2, and PKCs in various cell types, including SK-N-SH cells [18,20,21]. Moreover, galangin has been revealed to inhibit protein tyrosine kinase activity [48]. Galangin also attenuates MMP-9 expression by inhibiting PKC activity [29]. 12-O-tetradecanoylphorbol-13-acetate (TPA)-induced invasion and migration of HepG2 were inhibited by galangin through the attenuation of a PKC/ERK1/2 pathway [49]. In our study, we confirmed that galangin attenuated the MMP-9 expression induced by thrombin via the suppression of c-Src, Pyk2, and PKCα/βII or δ phosphorylation in SK-N-SH cells. However, we cannot exclude the possibility of galangin being an inhibitor of c-Src, Pyk2, or PKC catalytic activity.

Abnormal signal responses, such as Akt and mTOR, may implicate several brain inflammatory disorders. Thrombin has been reported to stimulate Akt and mTOR-dependent pathways to trigger pathophysiological processes in numerous cell types [50,51]. Galangin has been shown to induce autophagy by stimulating the phosphorylation of adenosine monophosphate-activated protein kinase (AMPK) and liver kinase B1 (LKB1) but inhibiting the phosphorylation of Akt and mTOR. The inhibition of AMPK activation suppresses the dephosphorylation of mTOR to block galangin-induced autophagy [52]. Another study has also revealed that galangin induces p53-mediated cell cycle arrest and the apoptotic process of nasopharyngeal carcinoma cells by suppressing the PI3K/Akt signaling pathway [53]. More recently, treatment of MG63 cells with galangin significantly inhibited the expression of cyclin D1 and MMP-2/9 via the suppression of PI3K and Akt phosphorylation [54]. In this study, we confirmed that these Akt/mTOR-dependent pathways play crucial roles in galangin reducing the MMP-9 expression induced by thrombin in SK-N-SH cells. Our findings showed that galangin inhibited the thrombin-stimulated phosphorylation of Akt and mTOR and attenuated MMP-9 expression in SK-N-SH cells. However, we cannot exclude the possibility of galangin being an inhibitor of Akt catalytic activity.

MAPKs pathways, including p42/p44 MAPK, p38 MAPK, and JNK1/2, participate in regulating the inflammatory reactions stimulated by proinflammatory factors, such as thrombin, cytokines, and chemokines [55,56,57]. In our recent studies, we have demonstrated that thrombin stimulates MAPK activation via PKCs and PI3K/Akt-dependent cascades, resulting in MMP-9 expression and cell migration in SK-N-SH cells [20,21]. Moreover, MAPKs have also been known to possess an important role in the regulation of the pathological processes of several CNS inflammatory disorders, such as stroke and AD [58,59,60,61]. Furthermore, several reports have shown that galangin elicits anti-inflammatory effects by inhibiting MAPK phosphorylation [62,63,64]. Galangin also attenuates airway remodeling by inhibiting transforming growth factor (TGF)-beta1-mediated reactive oxygen species (ROS) generation and MAPK/Akt phosphorylation in asthma [63]. In addition, galangin reduced phorbol-12-myristate-13-acetate (PMA)-induced MMP-9 expression by hampering NF-κB activation and JNK activation but not via inhibiting Erk1/2 or p38 MAPK activity in HT-1080 cells [29]. Moreover, the pharmacological properties of flavonoid compounds have been investigated in different types of cells. Several studies have demonstrated that flavonoids could exert an anti-cancer activity in hepatocellular carcinoma cells. For example, epigallocatechin-3-gallate (EGCG), a polyphenolic compound, has been shown to act as a potent inhibitor of the thrombin-PAR1/PAR4-p42/p44 MAPK invasive signaling axis in hepatocellular carcinoma cells [65]. In addition, flavonoid compounds including galangin have been demonstrated to exert their different anticancer effects via different intracellular signaling pathways and serve as antioxidants in various types of cells [66]. In this study, we confirmed that these MAPK-dependent signaling pathways were involved in galangin reducing the MMP-9 expression induced by thrombin in SK-N-SH cells. Our findings indicated that galangin inhibited the thrombin-stimulated phosphorylation of MAPKs to reduce MMP-9 expression in SK-N-SH cells. The differences in MAPKs between our study and others may be due to different experimental models and conditions. However, we cannot exclude the possibility of galangin being an inhibitor of MAPK catalytic activity.

Several transcription factors participate in the expression of inflammatory target genes associated with inflammation. Among these transcription factors, NF-κB, AP-1, and FoxO1 have central roles in regulating the expression of several genes, including MMP-9 associated with brain injury and inflammation during pathological events [62,67,68]. In our recent studies, we addressed that NF-κB- and AP1-dependent pathways participate in the MMP-9 expression induced by thrombin [20,21]. FoxO transcription factors have been revealed to regulate cell proliferation, apoptotic processes and cell cycle arrest. The mechanisms of thrombin-regulated smooth muscle cell proliferation are mediated via PI3K/Akt-dependent FoxO1 phosphorylation [69]. Moreover, galangin exerts its anti-inflammatory effects via the inhibition of NF-κB p65 and AP-1-dependent pathways to downregulate MMP-9 expression [29,62]. Our findings also showed that galangin inhibited the thrombin-stimulated phosphorylation of p65 and FoxO1, as well as p65 and c-Jun binding activities with the MMP-9 promoter to downregulate MMP-9 expression in SK-N-SH cells. Therefore, we confirmed that thrombin-induced MMP-9 expression was mediated via NF-κB, AP-1, and FoxO1, which were inhibited by galangin in SK-N-SH cells.

Our previous studies have shown that thrombin-induced MMP-9 expression is mediated via c-Src/Pyk2-dependent Akt and PLC/PKC-dependent signaling pathways, which stimulates MAPK-dependent activation of NF-κB and AP-1, leading to cell migration in SK-N-SH cells [20,21]. In this study, we extended the inhibitory effects of galangin on thrombin-induced MMP-9 expression and cell migration through the suppression of the activation of different protein kinases, including nRTKs, PKCs, Akt, mTOR, MAPKs, and transcription factors, such as NF-κB, AP-1, and FoxO1, in human SK-N-SH cells. Thus, the novelty of our study was more completely defining the detailed mechanisms by which galangin targeted signaling components and attenuated cell migration in a single type of human SK-N-SH cells. These results demonstrated that galangin attenuated the thrombin-induced MMP-9 expression via blocking the c-Src, Pyk2, Akt, mTOR, PKCα/βII or δ, p44/p42 MAPK, p38 MAPK, and JNK1/2-dependent NF-κB, AP-1, and FoxO1 cascade in SK-N-SH cells. In the present study, we only demonstrated that galangin had an inhibitory effect on the thrombin-stimulated phosphorylation of protein kinases and MMP-9 expression. We did not clarify the possibility of galangin directly or indirectly modulating the activities of these protein kinases. Thus, the inhibitory effects of galangin on targeting the signaling components are an important issue for further study.

## 4. Materials and Methods

### 4.1. Materials

The Dulbecco’s modified Eagle medium (DMEM)/F-12 medium, fetal bovine serum (FBS), and TRIzol were from Invitrogen (Carlsbad, CA, USA). The hybond membrane and enhanced chemiluminescence (ECL) western blotting detection system were from GE Healthcare Biosciences (Buckinghamshire, UK). The anti-phospho antibodies against p42/p44 MAPK (#9101), p38 MAPK (#9211), JNK1/2 (#4668), mTOR (#5536), Akt (#9271), c-Src (#2101), Pyk2 (#3291), PKCα/βII (#9375), PKCδ (#9374), p65 (#3031), FoxO1 (#9461), and mTOR (#2972) were from Cell Signaling (Danvers, MA, USA). The antibodies against p44 MAPK (sc-94), p42 MAPK (sc-154), p38 MAPK (sc-535), JNK1/3 (sc-474), JNK2 (sc-827), Akt (sc-8312), c-Src (sc-18), PKCα (sc-208), PKCδ (sc-213), and p65 (sc-7151) were from Santa Cruz (Santa Cruz, CA, USA). The antibody against GAPDH (#MCA-1D4) was from EnCor (Gainesville, FL, USA). The antibodies against Pyk2 (ab55358) and FoxO1A (ab52857) were from Abcam (Cambridge, MA, USA). Galangin (3,5,7-trihydroxy-2-phenyl-4H-1-benzopyran-4-one) was purchased from Cayman Chemicals (Ann Arbor, MI, USA). The thrombin, enzymes, and other chemicals were from Sigma (St. Louis, MO, USA). The SDS-PAGE reagents were from MDBio Inc (Taipei, Taiwan).

### 4.2. SK-N-SH Cell Cultures and Treatment

SK-N-SH cells, a human neuroblastoma cell line, were purchased from the American Type Culture Collection (Manassas, VA, USA) and cultured in DMEM/F12 supplemented with 10% FBS and antibiotics (100 U/mL penicillin G, 100 μg/mL streptomycin, and 250 ng/mL fungizone) at 37 °C in a humidified 5% CO_2_ atmosphere, as described previously [21]. When the cultures reached confluence (7 days), the cells were treated with 0.05% (*w*/*v*) trypsin/1 mM ethylenediaminetetraacetic acid (EDTA) for 3 min at 37°C. The cells were counted and diluted with DMEM/F12 to a final concentration of 2 × 10^5^ cells/mL. The cells were plated onto (1 mL/well) 12-well, (2 mL/well) 6-well culture plates, and (10 mL/dish) 10-cm culture dishes for these experiments. The medium was changed every 2–3 days. The experiments were performed with cells from passages 4–9. The cells were made quiescent by incubation in serum-free DMEM/F-12 for 24 h and then incubated with thrombin for the indicated time intervals. When galangin was used, it was added 1 h prior to the application of thrombin. These cells were incubated with either DMSO or various dosages of galangin alone for 24 h or 48 h. Galangin (up to 10 μM) had no significant effect on the cell viability determined by a 2,3-bis-(2-methoxy-4-nitro-5-sulfophenyl)-2H-tetrazolium-5-carboxanilide (XTT) assay kit (Figure S1).

### 4.3. Preparation of Cell Extracts and Western Blot Analysis

After incubation, the cells were then rapidly washed with ice-cold phosphate-buffered saline (PBS), scraped, and collected by centrifugation at 1,000 × g for 10 min, as described previously [21]. The collected whole cells were lysed with ice-cold lysis buffer containing: 25 mM Tris-HCl, pH 7.4, 25 mM NaCl, 25 mM NaF, 25 mM sodium pyrophosphate, 1 mM sodium vanadate, 2.5 mM EDTA, 2.5 mM ethylene glycol tetraacetic acid (EGTA), 0.05% Triton X-100, 0.5% SDS, 0.5% deoxycholate, 0.5% NP-40, 5 μg/mL leupeptin, 5 μg/mL aprotinin, and 1 mM phenylmethylsulphonyl fluoride (PMSF). The lysates were centrifuged at 45,000 × g for 1 h at 4 °C to yield the whole cell extract. The protein concentration was determined by using BCA reagents according to the instructions of the manufacturer. Samples from these supernatant fractions (30 μg protein) were denatured and subjected to SDS-PAGE using a 10% running gel. The proteins were transferred to nitrocellulose membrane and incubated successively at room temperature with 5% bovine serum albumin (BSA) in Tween-Tris buffered saline [(50 mM Tris-HCl, 150 mM NaCl, 0.05% Tween 20, pH 7.4)] for 1 h. The membranes were incubated overnight at 4 °C with their respective component antibody or anti-GAPDH antibody used at a dilution of 1:1000 in Tween-Tris buffered saline. The membranes were washed with Tween-Tris buffered saline 4 times for 5 min each and incubated with a 1:2000 dilution of anti-mouse or rabbit horseradish peroxidase antibody for 1 h. Following each incubation, the membrane was washed extensively with Tween-Tris buffered saline. The immunoreactive bands were detected by ECL reagents and captured by a UVP BioSpectrum 500 Imaging System (Upland, CA, USA). The image densitometry analysis was quantified by an UN-SCAN-IT gel software (Orem, UT, USA).

### 4.4. MMP Gelatin Zymography

After treatment, the culture media were collected and centrifuged at 1000 × g for 10 min at 4 °C to remove the cells and debris and then were electrophoretically separated on 10% SDS-polyacrylamide gels copolymerized with 1 mg/mL gelatin (Sigma-Aldrich) under non-reducing conditions, as described previously [21]. The gels were washed twice in 2.5% Triton X-100 to remove SDS and then incubated for 72 h with a developing buffer containing 50 mM Tris base, 40 mM HCl, 200 mM NaCl, 5 mM CaCl_2_, and 0.02% Brij-35 at 37 °C before staining with Coomassie Blue R-250. After incubation, the gels were stained in 30% methanol, 10% acetic acid, and 0.5% *w*/*v* Coomassie brilliant blue for 1 h followed by being destained to visualize the gelatinolytic bands (MMP-2/9) on a dark blue background. Mixed human MMP-2 and MMP-9 (Chemicon, Temecula CA, USA) were used as the gelatinase standards. Because cleaved MMPs are not reliably detectable, only pro-form zymogens were quantified.

### 4.5. RNA Extraction and Real-Time RT-PCR Analysis

Total RNA from SK-N-SH cells treated by the indicated conditions was extracted using a TRIzol reagent according to the protocol of the manufacturer. The mRNA was reverse-transcribed into cDNA using 2 μg of total RNA and random hexamers as primers in a final volume of 20 μL (5 μg/μL random hexamers, 1 mM dNTPs, 2 U/μL RNasin, and 10 U/μL Moloney murine leukemia virus reverse transcriptase).Then, the cDNA was analyzed by real-time PCR, as described previously [21]. Real-time PCR was performed using SYBR Green PCR reagents (Applied Biosystems, Branchburg, NJ, USA) and primers specific for MMP-9 and GAPDH mRNAs. The level of MMP-9 was determined by normalizing to GAPDH expression. The primers were: MMP-9:5′-CGATGCCTGCAACGTGAAC-3′ (sense) and 5′-AGAGCCGCTCCTCAAAGACC-3′ (anti-sense); GAPDH: 5′-GCCAGCCGAGCCACAT-3′ (sense) and 5′-CTTTACCAGAGTTAAAAGCAGCCC-3′ (anti-sense).

### 4.6. Human MMP-9 Promoter Cloning, Transient Transfection, and Promoter Activity Assays

A 710 bp (−720 to −11) segment from the 5′-promoter region of the MMP-9 gene was cloned as previously described [70]. Briefly, a 0.71 kb segment at the 5′-flanking region of the human MMP-9 gene was amplified by PCR using specific primers from the human MMP-9 gene (accession no. D10051): 5′-ACATTTGCCCGAGCTCCTGAAG (forward/SacI) and 5′-AGGGGCTGCCAGAAGCTTATGGT (reverse/HindIII). The pGL3-Basic vector, containing a polyadenylation signal upstream from the luciferase gene, was used to construct the expression vectors by subcloning PCR-amplified DNA of the MMP-9 promoter into the SacI/HindIII site of the pGL3-Basic vector. The PCR products (pGL3-MMP-9) were confirmed by their sizes, as determined by electrophoresis, and by DNA sequencing. MMP-9-luc reporter construct plasmids were transiently transfected at a concentration of 0.8 μg/mL, and the control pGal encoding for β-galactosidase presented at 0.2 μg/mL to normalize the transfection efficiency. To assess promoter activity, the cells were collected and disrupted by sonication in lysis buffer (25 mM Tris, pH 7.8, 2 mM EDTA, 1% Triton X-100, and 10% glycerol). After centrifugation, aliquots of the supernatants were collected, and the MMP-9-luc luciferase activities were determined by using a luciferase assay system (Abcam, Cambridge, UK) according to the manufacturer’s instructions. Detected firefly luciferase activities were standardized with β-galactosidase activity.

### 4.7. Cell Migration Assay

When they reached confluence, the monolayer cells were manually scratched with a pipette tip to create a straight scratch wound in the center of the well with a bright and clear field (~2 mm), as described previously [21]. The detached cells were removed by washing the cells once with PBS. Serum-free DMEM/F-12 medium with or without thrombin was added to each well as indicated after pretreatment with galangin for 1 h, containing a DNA synthesis inhibitor hydroxyurea (10 μM) during the period of experiments. Images of migratory cells from the scratch boundary were observed and acquired at 0 and 48 h with a digital camera coupled with a light microscope (Olympus, Tokyo, Japan). The numbers of migratory cells were counted from the resulting 4-phase images for each point and then averaged for each experimental condition. The data presented were summarized from three separate assays.

### 4.8. Chromatin Immunoprecipitation Assay

To detect the in vivo association of the nuclear proteins with the human MMP-9 promoter, chromatin immunoprecipitation analysis was conducted as previously described [71]. Briefly, SK-N-SH cells were cross-linked with 1% formaldehyde for 10 min at 37 °C and washed thrice with ice-cold PBS containing 1 mM phenylmethylsulphonyl fluoride (PMSF) and 1% aprotinin. The cell lysates were prepared using a SDS-lysis buffer (1% SDS, 5mM EDTA, 1mM PMSF, 50 mM Tris-HCl) and were sonicated at 4 °C until the DNA size became 200–300 base pairs. After soluble chromatin was precleared by incubation with sheared salmon sperm DNA-protein agarose A, one portion of the sample was as DNA input control; the other supernatant was immunoprecipitated without (control) or with anti-p65, anti-c-Jun antibody, and protein A beads. After washing and elution, the precipitates were heated overnight at 65 °C to reverse the cross-linking of the DNA and proteins. The DNA fragments were purified by phenol-chloroform extraction and ethanol precipitation. The purified DNA was subjected to PCR amplification using the primers specific for the region (−666 ~ −308, Accession NO: NC000020) containing the distal AP-1 binding site (−539 to −533) and NF-κB binding site (−605 to −595) present in the MMP-9 promoter region, sense primer (NF-κB): 5′-GCTACTGTCCCCTTTACTGCCC-3′; antisense primer (NF-κB): 5’-GGGGGATGAAGCTGGAGGG-3′, sense primer (AP-1): 5′-GCTACTGTCCCCTTTACTGCCC-3′; antisense primer (AP-1): 5′-TCTTTGACTCAGCTTCCTCTCC-3′. The PCR fragments were analyzed on 3% agarose in 1× TAE gel containing ethidium bromide, and the size (340 bp of NF-κB and 139 bp of AP-1) was compared to a molecular weight marker.

## 5. Statistical Analysis of Data

All the data were expressed as the mean or mean ± SEM of five individual experiments performed in duplicate or triplicate. The significance of the difference between the two groups was determined by paired two-tailed Student’s t-test for western blot data. All the others statistical analyses were a comparison of multiple groups, a GraphPad Prism Program (GraphPad, San Diego, CA, USA) by one-way analysis of variance (ANOVA) followed by Tukey’s post-hoc test was used. A *p* < 0.05 value was considered significant.

## 6. Conclusions

In the central nervous system, MMP-9 is involved in numerous pathophysiological responses, such as ECM degradation, tissue remodeling, neurite outgrowth, and injury repair [44]. Moreover, upregulation of MMP-9 may be implicated in the pathological processes of brain disorders, including stroke, AD, multiple sclerosis, and malignant glioma [72]. These findings of galangin inhibiting the thrombin-induced MMP-9 expression in SK-N-SH cells indicate that galangin might be a potential candidate for the therapeutic agents of brain inflammatory and degenerative diseases. However, the limitations of this study were that there was no evidence to clarify the inhibitory effects of galangin in vivo. Moreover, the anti-inflammatory molecules induced by galagin to protect against brain inflammation are still unknown. Therefore, it is important to further explore the effect of galagin in vivo and investigate which molecules participate in its anti-inflammatory effects.

## Supplementary Materials

Supplementary materials can be found at http://www.mdpi.com/1422-0067/19/12/4084/s1. Figure S1: Effect of galangin on SK-N-SH cell cyto-toxicity.

## Abbreviations

AD	Alzheimer’s disease
AMPK	Adenosine monophosphate-activated protein kinase
BBB	Blood–brain barrier
ECL	Enhanced chemiluminescence
ECM	Extracellular matrix
FBS	Fetal bovine serum
LKB1	Liver kinase B1
MMPs	Matrix metalloproteinases
NF-κB	Nuclear factor-κB
nRTKs	non-receptor tyrosine receptor kinases
PMSF	Phenylmethylsulphonyl fluoride
XTT	2,3-bis-(2-methoxy-4-nitro-5-sulfophenyl)-2H-tetrazolium-5-carboxanilide
