# Supplementary material for: Galangin Inhibits Thrombin-Induced MMP-9 Expression in SK-N-SH Cells via Protein Kinase-Dependent NF-κB Phosphorylation
Source: Int J Mol Sci. 2018 Dec 17;19(12):4084. doi: 10.3390/ijms19124084 (PMC6321481; doi:10.3390/ijms19124084)
Supplement: Supplementary file 1 [file ijms-19-04084-s001.zip › ijms-389288-supplementary.pptx]

## Slide 1
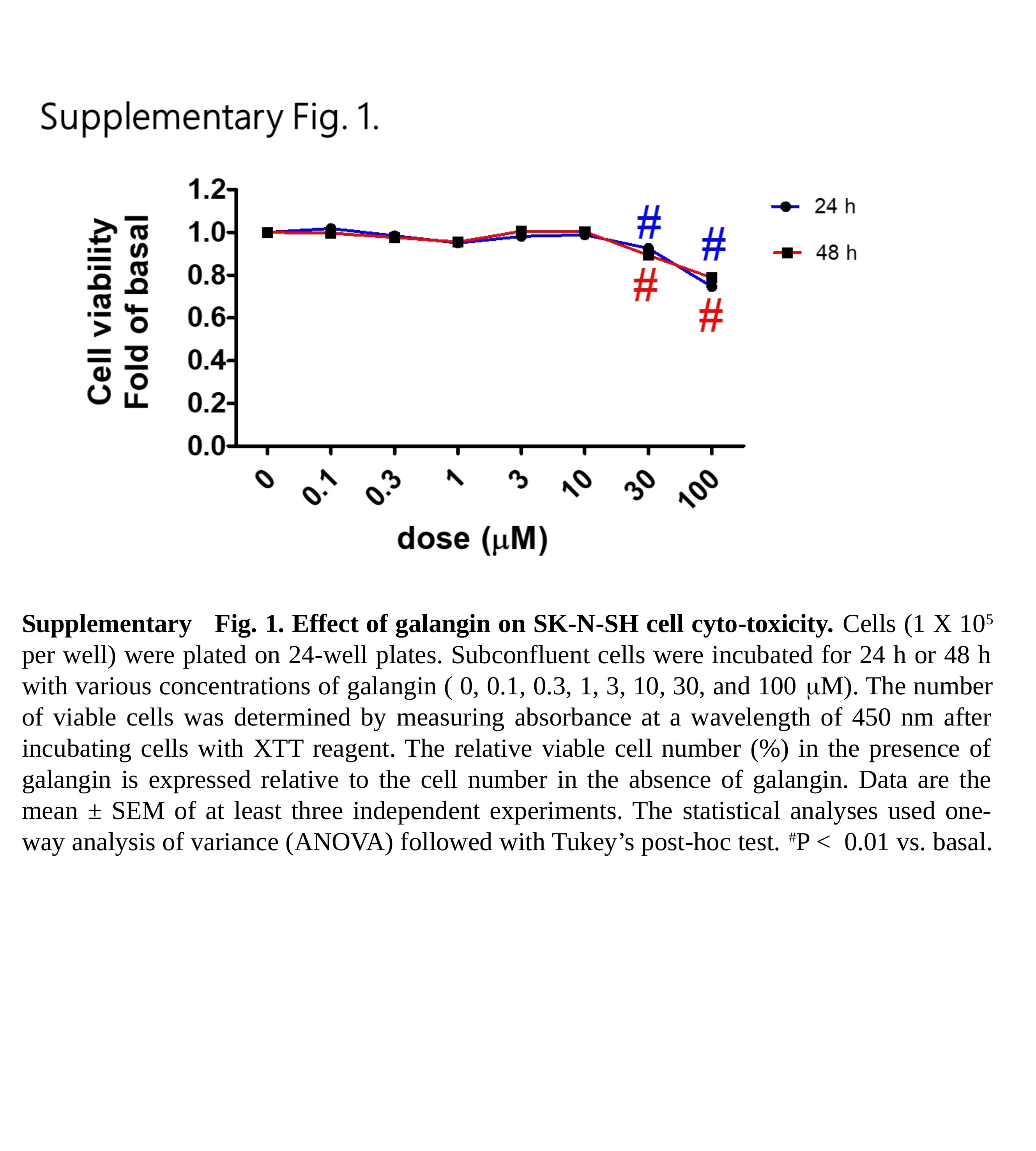

Supplementary Fig. 1. Effect of galangin on SK-N-SH cell cyto-toxicity. Cells (1 Х 105 per well) were plated on 24-well plates. Subconfluent cells were incubated for 24 h or 48 h with various concentrations of galangin ( 0, 0.1, 0.3, 1, 3, 10, 30, and 100 μM). The number of viable cells was determined by measuring absorbance at a wavelength of 450 nm after incubating cells with XTT reagent. The relative viable cell number (%) in the presence of galangin is expressed relative to the cell number in the absence of galangin. Data are the mean ± SEM of at least three independent experiments. The statistical analyses used one-way analysis of variance (ANOVA) followed with Tukey’s post-hoc test. #P < 0.01 vs. basal.
